# Fus2Net: a novel Convolutional Neural Network for classification of benign and malignant breast tumor in ultrasound images

**DOI:** 10.1186/s12938-021-00950-z

**Published:** 2021-11-18

**Authors:** He Ma, Ronghui Tian, Hong Li, Hang Sun, Guoxiu Lu, Ruibo Liu, Zhiguo Wang

**Affiliations:** 1 College of Medicine and Biological Information Engineering, Northeastern University, Shenyang, Liaoning 110169 China; 2 Department of Nuclear Medicine, General Hospital of Northern Theater Command, No. 83 Wenhua Road, Shenhe District, Shenyang, Liaoning 110016 China; 3 Key Laboratory of Intelligent Computing in Medical Image, Ministry of Education, Shenyang, China

**Keywords:** Convolutional Neural Network, Deep learning, Data augmentation, Breast ultrasound tumor images, Classification

## Abstract

**Background:**

The rapid development of artificial intelligence technology has improved the capability of automatic breast cancer diagnosis, compared to traditional machine learning methods. Convolutional Neural Network (CNN) can automatically select high efficiency features, which helps to improve the level of computer-aided diagnosis (CAD). It can improve the performance of distinguishing benign and malignant breast ultrasound (BUS) tumor images, making rapid breast tumor screening possible.

**Results:**

The classification model was evaluated with a different dataset of 100 BUS tumor images (50 benign cases and 50 malignant cases), which was not used in network training. Evaluation indicators include accuracy, sensitivity, specificity, and area under curve (AUC) value. The results in the Fus2Net model had an accuracy of 92%, the sensitivity reached 95.65%, the specificity reached 88.89%, and the AUC value reached 0.97 for classifying BUS tumor images.

**Conclusions:**

The experiment compared the existing CNN-categorized architecture, and the Fus2Net architecture we customed has more advantages in a comprehensive performance. The obtained results demonstrated that the Fus2Net classification method we proposed can better assist radiologists in the diagnosis of benign and malignant BUS tumor images.

**Methods:**

The existing public datasets are small and the amount of data suffer from the balance issue. In this paper, we provide a relatively larger dataset with a total of 1052 ultrasound images, including 696 benign images and 356 malignant images, which were collected from a local hospital. We proposed a novel CNN named Fus2Net for the benign and malignant classification of BUS tumor images and it contains two self-designed feature extraction modules. To evaluate how the classifier generalizes on the experimental dataset, we employed the training set (646 benign cases and 306 malignant cases) for tenfold cross-validation. Meanwhile, to solve the balance of the dataset, the training data were augmented before being fed into the Fus2Net. In the experiment, we used hyperparameter fine-tuning and regularization technology to make the Fus2Net convergence.

## Background

The most common malignant tumor occurring in Chinese women is breast cancer and its incidence rate is increasing annually. Numerous studies have confirmed that breast cancer screening is the most effective way to improve the early diagnosis rate and survival rate of breast cancer patients. Early breast cancer is a curable disease, and early treatment is the best way of raising the survival rate of breast cancer [1]. Developed countries have implemented breast cancer screening guidelines early, and the 5-year survival rate of breast cancer has been increased to 89%. With the development of China’s society and economy, there is an urgent need to increase the level of breast cancer prevention in women. Compared with Western women, Chinese women have denser breasts. In addition, the peak age of breast cancer in Chinese women is between 40-50 years, which is earlier than women in Western countries [2]. Large-scale and rapid screening of benign and malignant breasts based on the computer-aided diagnosis (CAD) system has attracted more attention from researchers in recent years.

At present, normal breast cancer examination methods include mammography, ultrasonography, magnetic resonance imaging, positron emission tomography, and biopsy [3]. Among them, ultrasonography has the characteristics of cost-effective, radiation-free, and small side effects, and is widely used in the early screening of breast cancer. Since the acoustic properties of normal tissues and cancer tissues are very similar, it is difficult for an experienced radiologist to distinguish between them. Therefore, CAD systems based on machine learning methods have been applied to ultrasound diagnosis.

Researchers have applied a variety of algorithms for feature selection to locate and classify breast lesions in recent years. The authors studied three backpropagation artificial neural network algorithms based on gradient descent and evaluated its performance in distinguishing the breast ultrasound (BUS) tumors as benign and malignant [4]. Results for classification of the 57 extracted texture and shape features giving the highest classification accuracy of 84.6%. Another literature [5] proposed a watershed method for semi-automatic tumor segmentation. After extracting a set of 855 features including shape or texture from each tumor area, a Bayesian Automatic Relevance Detection (ARD) was used to reduce the feature and dimensionality. The evaluation using eightfold cross-validation on a dataset of 104 BUS tumor images, an accuracy with 97.12% was achieved. Chen *et al.* [6] proposed a bi-clustering mining method to acquire high-level features. A total of 238 tumors instances (including 115 benign cases and 123 malignant cases) were classified with two hidden layers neural networks and obtained the accuracy, sensitivity, specificity with 96.1, 96.7, 95.7%, respectively. Although the above research achieved some satisfactory results, the datasets were either too small or from different ultrasonic machines, making it difficult to implement generalization. In a few attempts to locate and classify tumors [7, 8], the overall performance of automatically locating regions of interest and classifying breast lesions employing different Convolutional Neural Network (CNN) architecture has been improved, comparing with traditional classification algorithms [9].

Deep Learning (DL) performs better than traditional machine learning algorithms in object classification [10]. In recent years, DL methods using Convolutional Neural Networks (CNNs) have achieved significant advantages in the field of medical image analysis [11]. Some researchers have used the well-known CNN architecture to classify benign and malignant BUS tumor images [12]. Due to the limited public BUS dataset, the Transfer Learning (TL) method using the pre-trained classification model is feasible [13, 14]. TL methods use the well-known CNN classification model as a feature extractor for automatic feature selection of images. After the output bottleneck layer, a multi-layer neural network is added to classify features, which is called a customed classifier. To deal with BUS images, it is also effective to build a CNN for specific classification tasks [15–17]. Generally, researchers will augment the collected toy datasets. Data augmentation can achieve more complex representations of data, reduce the difference between the training set and test set, and allow CNNs to better learn the data distribution on the whole dataset [18–20]. Shallow convolutional layers can extract low-dimensional abstract features, such as edges and spots, etc. Deeper convolutional layers extract higher dimensional abstract features, which is crucial for specific classification tasks. To improve the classification performance of the model, CNNs need to take into account the characteristics of different dimensions at the same time. Using a pre-trained model based on natural image training, the TL method often needs to fine-tune for specific tasks. Equally, the conventional stacked convolutional layer has such an issue in multi-scale expression capabilities.

In this paper, a novel CNN named Fus2Net for specific classification tasks was proposed. Different from conventional CNNs that are executed layer-wisely, we have exploited the multi-scale expression potential of CNN at a more granular level. In addition, the low-dimensional and high-dimensional feature maps are fused before being input to the fully connected layer, combining the characteristics of different levels.

## Results

The Fus2Net was implemented through the Keras module of TensorFlow2.0 and was trained on the Windows 10 professional system using Nvidia 1080Ti 16GB with CUDA 3584 cores, GPU. Other relevant hardware information includes: Intel(R) Core (TM) i7-8700k CPU 3.70GHz, 16.0GB RAM, Anaconda Jupyter notebook IDE and Python computer programming language.

Considering the impact of the image format on the classification network, we used the original three-channel RGB format BUS image and the single-channel BUS grayscale image to conduct a comparative experiment. Table 1 shows the classification performance metrics of single-channel and three-channel BUS images. The results show that three-channel images have advantages over single-channel images in terms of classification performance.

**Table 1:** The performance metrics of single-channel and three-channel BUS tumor images

	Accuracy	Sensitivity	Specificity	Precision	AUC
One channel	81.31%	78.51%	85.42%	84.57%	0.86
Three channels	**85.08%**	**82.01%**	**88.34%**	**86.93%**	**0.89**

The bold value in the table indicates this method (in row) outforms others regarding this specific metric (in column)

**Fig. 1:**
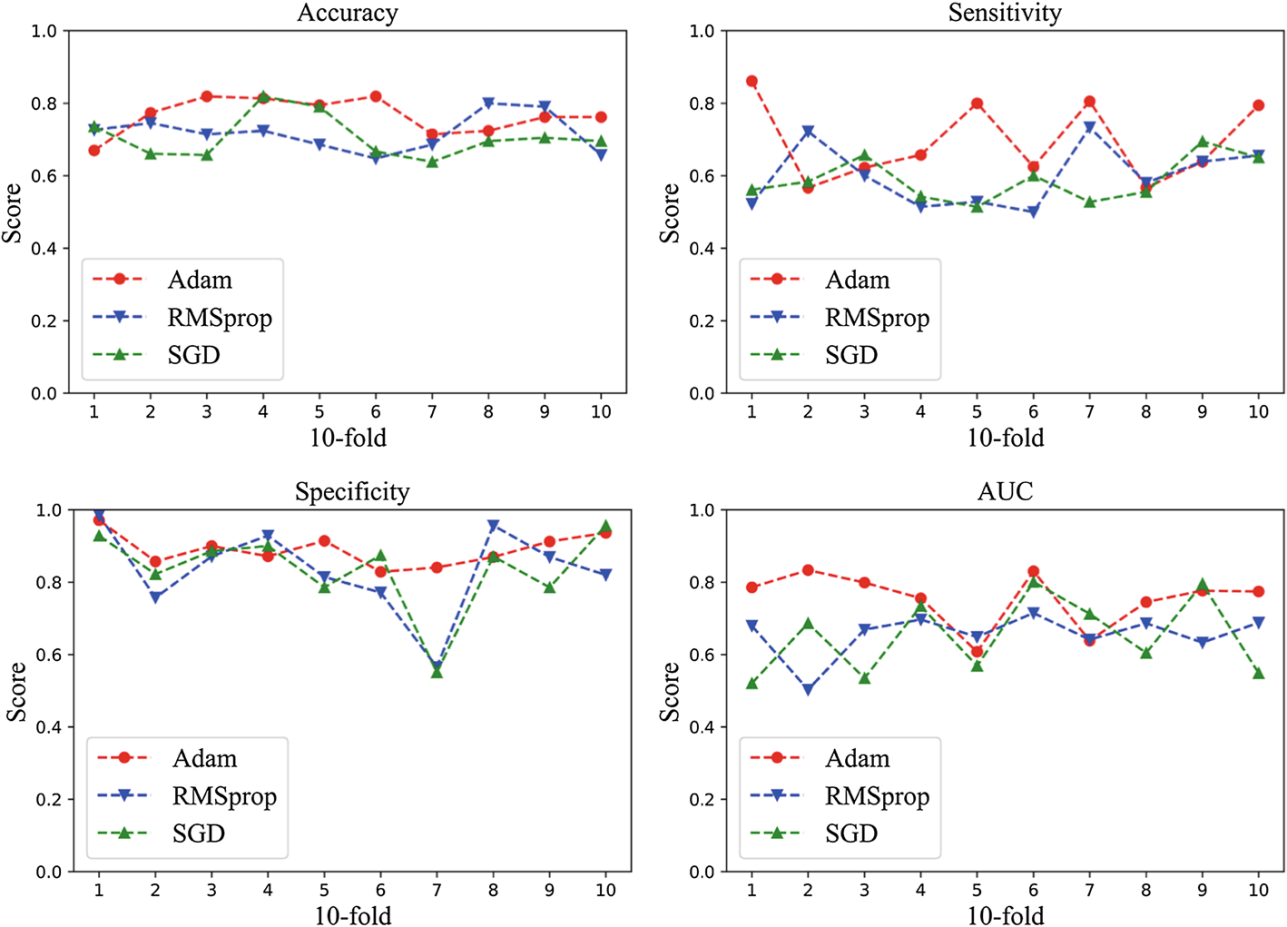
Performance metrics of the Fus2Net using Adam, RMSprop and SGD optimizers in accuracy, sensitivity, specificity, AUC

We verify the performance of different optimizers on BUS tumor images without preprocessing as our candidates. Fig. 1 summarizes the classification performance metrics of Fus2Net using Adam, RMSprop and SGD optimizers in tenfold cross-validation. The results demonstrate that among the three common optimization algorithms, by calculating the mean values of the three optimizers in accuracy, specificity, sensitivity and AUC value, Adam optimizer is $5\%$ higher than RMSprop and SGD, which indicates the advantages of Adam optimizer in BUS tumor image classification. In general, the Adam optimization algorithm is superior to RMSprop and SGD in BUS tumor image classification.

After determining the input format of the BUS tumor image and the optimizer of training the Fus2Net, data augmentation was used to slow down overfitting and raise the generalization ability of classification model, and regularization technique was used to avoid overfitting. We perform tenfold cross-validation on the training data and calculate the mean to compare the performance metrics of Fus2Net after applying image augmentation and regularization techniques, as shown in Table 2. The results demonstrate that the image augmentation and regularization technology improve the performance metrics of Fus2Net.

**Table 2:** The performance metrics after using image augmentation, L2 regularization, and dropout

	Accuracy	Sensitivity	Specificity	Precision	AUC
Image augmentation	88.81%	92.05%	82.58%	85.37%	0.91
Image augmentation and L2 regularization and dropout	**93.25%**	**94.19%**	**88.57%**	**91.94%**	**0.97**

The bold value in the table indicates this method (in row) outforms others regarding this specific metric (in column)

**Fig. 2:**
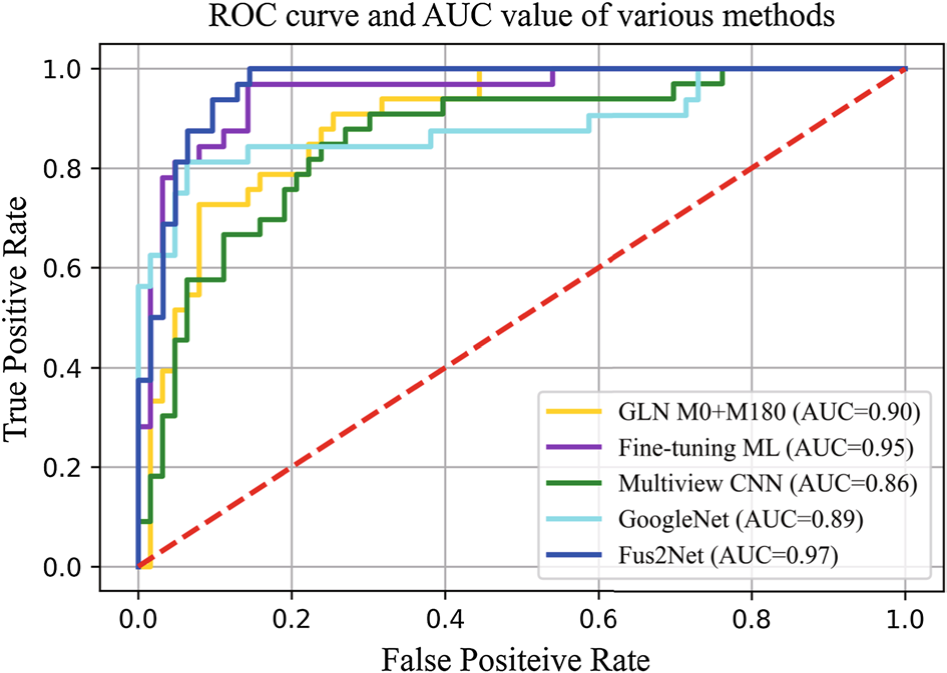
The ROC curve, and the AUC value of our proposed Fus2Net, and four existing CNN-based methods

To evaluate the application of the Fus2Net classification model in real scenarios, we tested 100 BUS tumor images that did not participate in the training. Meanwhile, we compared the existing classification methods of BUS tumor images based on deep learning. Table 3 shows the test results of our proposed Fus2Net and existing methods on 100 BUS tumor images. Fig. 2 presents the ROC curve of the BUS tumor image classification method. Experiments demonstrate that, compared with the existing CNN methods, our proposed Fus2Net has advantages in classification and evaluation metrics such as accuracy, sensitivity, specificity, and AUC value.

**Table 3:** The performance metrics of the Fus2Net and other four methods

	Accuracy	Sensitivity	Specificity	Precision	AUC
GLN M0$+$M80 [16]	85%	88.89%	81.82%	80%	0.90
Fine-tuning ML [12]	88%	85.19%	**91.30%**	**92%**	0.95
Multiview CNN [13]	78%	83.33%	74.14%	70%	0.86
GoogleNet [11]	82%	80.77%	83.33%	84%	0.89
Fus2Net	**92%**	**95.65%**	88.89%	88%	**0.97**

The bold value in the table indicates this method (in row) outforms others regarding this specific metric (in column)

## Discussion

The Fus2Net we designed to acquire a fine recognition performance in the automatic classification of benign and malignant BUS tumor images. The classification architecture of Fus2Net is shown in Fig. 7. Augmented training data simulate actual clinical ultrasound images and improve the robustness of the Fus2Net. Using 100 BUS tumor images without participating in training, we tested Fus2Net and four existing CNN-based classification methods of BUS tumor images. Experiments show that the Fus2Net performs better in performance metrics. Among all the methods, Fus2Net performs best in accuracy, AUC, and sensitivity. Only the fine-tuning ML method is slightly higher than our method in specificity. In terms of overall performance, the performance of the fine-tuning ML method is closest to our method, and the worst performance is the Multiview CNN method. In terms of accuracy, Fus2Net reached 0.92, the highest among all methods. In terms of sensitivity, Fus2Net reached 95.65%, which is higher than the fine-tuning ML method, indicating that the CNN we designed has a higher classification accuracy for malignant tumors. In terms of specificity, Fus2Net reached 88.89%, which is 3% lower than the fine-tuning ML method, indicating that the CNN we designed has lower classification accuracy for benign tumors. In terms of the most critical indicator AUC value, Fus2Net reached 0.96, higher than the other four CNN-based classification methods.

**Fig. 3:**
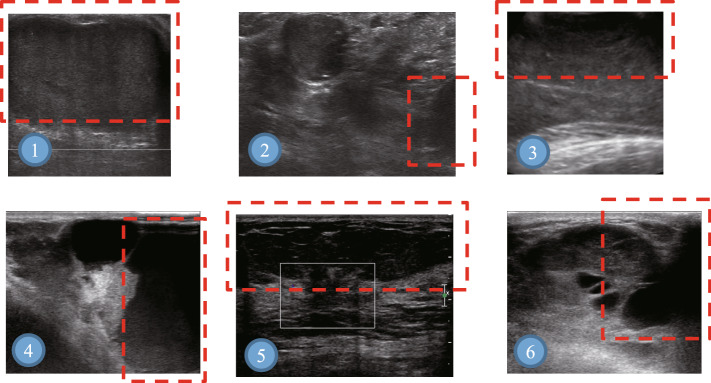
Samples misclassified by Fus2Net. Among them,benign images: 1, 2, 3; malignant images: 4, 5, 6

We attempt to analyze the BUS tumor images misclassified by Fus2Net. Misclassified images have tumor boundaries that exceed the size of the image, which makes Fus2Net unable to perform convolution calculations on complete tumors, as shown in the red dotted mark in Fig. 3 The convolution kernel needs to perform convolution calculations on the complete object so that the bottleneck layer can better represent the image category. In addition, in the process of image acquisition, if standardized acquisition can be carried out, it is of great significance for the automatic classification of benign and malignant BUS tumor images. In the next stage, we will continue to communicate with partner hospitals to collect higher quality BUS tumor images and further improve the level of automated auxiliary diagnosis.

The fusion of feature extraction modules is the core innovation of Fus2Net. In terms of architecture, the properties of the convolution kernel inside a single feature extraction module are equally important. The Block 1 module has three branches, and the number and size of the convolution kernels of each branch are obtained through fine-tuning of the architecture, to extract features of the BUS tumor images more efficiently. The 1 x 1 convolution kernel can achieve feature dimensionality reduction, reduce the number of parameters, and improve the representation capabilities of the generated feature maps. The 1 x 7 and 7 x 1 convolution kernels deepen the depth of Fus2Net and increase its nonlinearity. The two branches in Block 1 use the maximum pooling and average pooling operations, respectively. The introduction of the pooling layer not only reduces the parameters, prevents Fus2Net from overfitting, but also improves the generalization ability of the classification model. The first half of the Block 2 module is similar to the Block 1 module, and the second half introduces a scale residual unit. The scale residual is used to eliminate the influence of network depth on the performance of Fus2Net and strengthen the feature expression in the hidden layer. In addition, the addition operation in Block 2 combines low-dimensional and high-dimensional feature maps to improve the multi-scale expression ability of the Fus2Net.

In BUS tumor image processing, we have augmented the original image. Image augmentation makes the images participating in the training more robust, and the generated classification model is more general, which significantly improves the classification performance of the model on the test set. The traditional ultrasound image enhancement method is based on pixels for a single image. Different from that, the augmentation operations such as shift, flip, and shear make the data richer without changing the pixel difference of the original image. For DL technology, image augmentation has more advantages than single image enhancement.

The experiments showed that a large amount of training data is still a powerful tool for DL technology. In future research, we can seek more experimental data, and continue to optimize the model by considering transfer learning. According to the existing research results, transfer learning has better performance for small-scale datasets. Moreover, the model used in transfer learning is based on open dataset training. In the following research, we can use the multi-modality images of different organs to train the model and then perform the transfer training for BUS tumor images to achieve better results. The main reason is that there are big differences between medical data and public datasets of natural scenes. The model trained with other data belonging to medical images can reduce the differences between classes. On this basis, the use of TL technology may have a better result.

## Conclusions

In this study, we proposed the Fus2Net to distinguish benign and malignant BUS tumor images. The experiment is based on the training data to perform tenfold cross-validation to select the optimizer, verify the effect of regularization, and adjust the hyperparameters for Fus2Net. The classification results of Fus2Net on 100 BUS tumors without training showed that the accuracy was 0.92, the sensitivity was 95.65%, the specificity was 88.89%, and the AUC was 0.97. The Fus2Net classification framework we proposed has a better auxiliary effect for radiologists to distinguish benign and malignant BUS tumors and is superior to existing methods.

## Methods

In this paper, we proposed Fus2Net architecture for distinguishing benign and malignant tumors in BUS images. Training a CNN model requires large-scale data [21]. However, the experimental data collected are limited. Due to this reason, we applied image enhancement technology to augment the dataset, which proved to be effective for CNN training [22]. During CNN training, different regularization techniques are used to reduce overfitting and hyperparameter adjustments to improve the classification performance of the Fus2Net model.

**Fig. 4:**
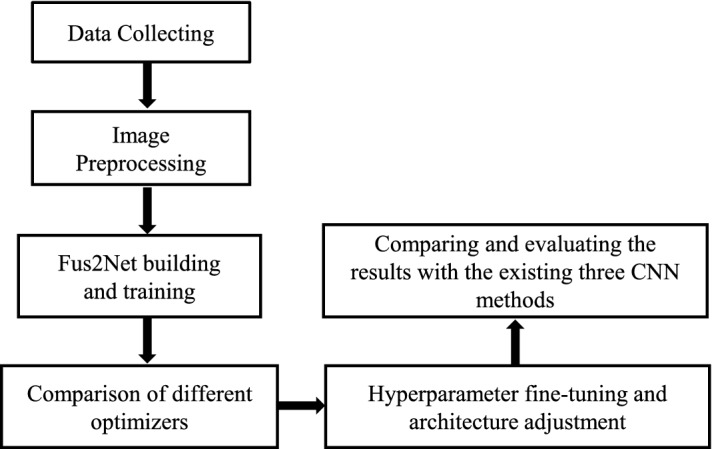
Flowchart of our proposed method

The proposed classification process for BUS tumor images in this study comprises: data collection, image preprocessing, creating and training the CNN, comparison of different optimizers and loss functions, hyperparameter adjustment and architecture fine-tuning, comparison of different CNN classification methods and results in the evaluation. Fig. 4 shows a block diagram of these steps.

### Clinical dataset

The dataset used in the experiment was collected from the local hospital. The entire dataset comprised 1052 images, which include 696 benign solid cysts and 356 malignant solid cysts. They are captured from different devices, such as GE LOGIQ E9 and PHILIPS EPIQ5. The patient information in all images is hidden. Each image is labeled as benign or malignant through biopsy and serves as the ground truth for training data. The single-data format is a three-channel PNG file with a depth resolution of 24 bits and a resolution of 775 x 580 pixels.We reserved 100 cases (50 benign and 50 malignant) for model evaluation, and 952 cases were used as training set to fit the classification model.

For our retrospective study, the informed consent for data usage was approved by the Medical Ethics Committee of the First Hospital of China Medical University.

### Data preprocessing

The preprocessing stages before the images are input to Fus2Net are listed as follows: balance of benign and malignant data, image resize, data augmentation, and image standardization.

Training Fus2Net used 646 benign images and 306 malignant images. To balance the number of benign and malignant images, we randomly selected 170 malignant images to flip horizontally and vertically. In the end, the number of malignant images increased to 646, and the benign and malignant data were balanced.

Different from multi-layer neural networks, the input of CNNs is a pixel matrix of the two-dimensional image. The size of the image determines the training time of the CNN and the memory required for processing. Generally, the well-known CNNs architecture chooses images with a size of 224 x 224 or 320 x 320 pixels. Our experiment is conducted on a GPU with stronger computing power while retaining as much image information as possible. We use bilinear interpolation to resize the images to 299 x 299 pixels.

**Fig. 5:**
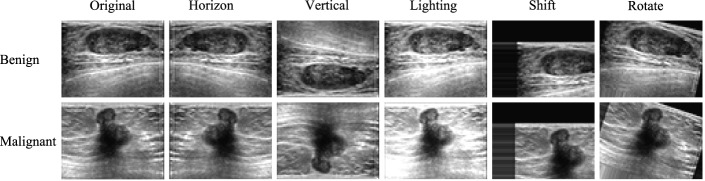
Samples of image augmentation on the benign and malignant

To avoid Fus2Net from overfitting during training, we performed image data augmentation processing on the balanced and resized images [18]. The augmentation methods used involve rotation, lighting, shift, etc. Augmented examples of a single image in the experiment are presented in Fig. 5.

The different distribution of training data will reduce the training speed of CNN and bring difficulties to model convergence. Before the training images are input into Fus2Net, we performed zero-mean normalization processing on them. After that, the image features and the relationship between the features will not change with the standardization. Image standardization can make CNN easier to learn. Equation 1 is the definition of image zero-mean standardization:

1 $$\begin{aligned} x^{*}=\frac{x-\mu }{\sigma }, \end{aligned}$$

where *x* is the original image, $x^{*}$ is the resulting image, $\mu$ is the mean value of the image, and $\sigma$ is the standard deviation of the image.

**Fig. 6:**
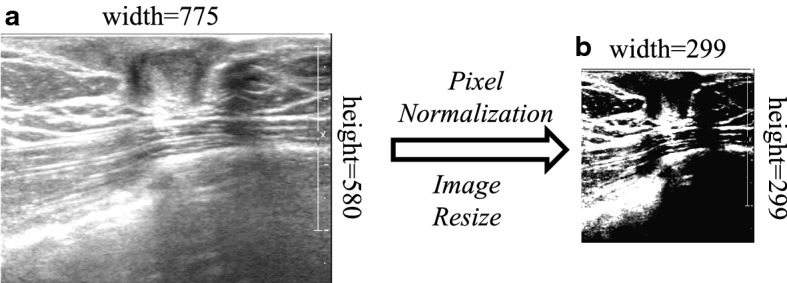
The effect of the original image after preprocessing on a sample: a original image, b resulted image through image preprocessing

The preprocessing of the data enhances the representation of the image and also improves the generalization of the CNN model. Fig. 6 shows the original image and the final image resulting from data preprocessing.

### Fus2Net framework

**Fig. 7:**
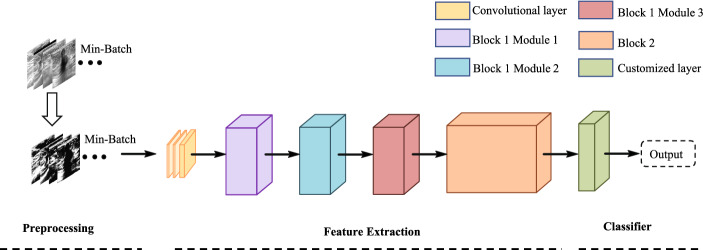
The classification architecture of the Fus2Net

The Fus2Net framework proposed in this paper is shown in Fig. 7. The feature extraction part is composed of three convolutional types: three basic convolutional layers, Block 1 module and Block 2 module. Each convolution type has filters of different sizes and numbers. There are mainly four sizes of filters used: 1 x 1, 3 x 3, 1 x 7, 7 x 1, and the strides size is 1 x 1 or 2 x 2. After the convolutional layer, the bottleneck feature is output as the input of the customized classifier, and the classification prediction probability is obtained under the action of the activation function. The customization layer is composed of two fully connected layers. The final fully connected layer has only two neurons, which directly classifies the features of the dense layer.

The basic convolution layer uses the 3 x 3 size convolution kernel. It occupies a dominant position in all CNN models and can improve the performance of the neural network to a certain extent [23, 24]. In addition, the first convolutional layer uses a stride size of 2 x 2, and the other two convolutional layers use the same stride size of 1 x 1 acquiescently. After each convolutional layer, a Rectified Linear Unit (ReLU) is used, which avoids the issue of vanishing gradient [25].

**Fig. 8:**
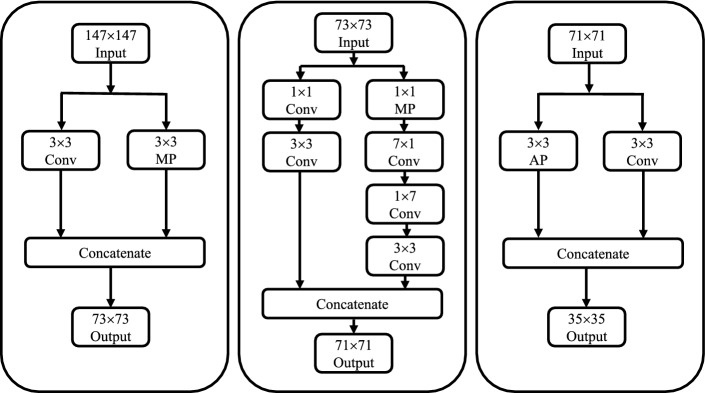
Three architectures of Block 1: Module 1, Module2, Module 3

Block 1 includes three modules, which increase the multi-scale representation capability of Fus2Net [26]. Each module has two branches, and the size and number of filters on each branch are different. The two branches of Module 1 use the 3 x 3 convolutional layer and the maximum pooling layer, respectively. The two branches of Module 2 use six convolutional layers that contain all types. Module 3 is similar to Module 1 and replaces the maximum pooling layer with the average pooling layer. The feature maps of all branches in Block 1 are concatenated and output. Fig. 8 presents the complete architecture of Block 1.

**Fig. 9:**
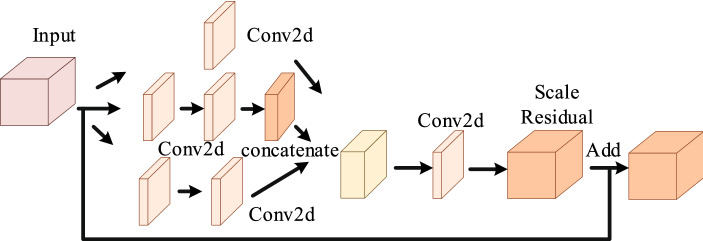
The detailed structure of the Block 2

Block 2 module is consisted of four branches. Convolutional layers are used in the three branches, and feature maps are concatenated. Then the feature map passes through a 1 x 1 convolutional layer and scale residual unit [27]. For the fourth branch, we add the adjusted input features and the features of the scaled residual unit. Fig. 9 is the structure of Block 2.

Table 4 summarizes the parameters of the main layers of the Fus2Net.

**Table 4:** The detailed parameters of the Fus2Net

	$\text{Layer' s name}$	$\text{Input size}$	$\text{Output size}$	$\text{Filter size}$	Strides
1	Input_Layer	(299, 299, 3)	(299, 299, 3)	None	None
2	Conv2d_1	(299, 299, 3)	(149, 149, 32)	(3, 3)	(2, 2)
3	Conv2d_2	(149, 149, 3)	(147, 147, 32)	(3, 3)	(1, 1)
4	Conv2d_3	(147, 147, 3)	(147, 147, 64)	(3, 3)	(1, 1)
		Block 1 module 1			
5	Max_pooling2d	(147, 147, 64)	(73, 73, 64)	(3, 3)	(2, 2)
6	Conv2d_4	(147, 147, 64)	(73, 73, 96)	(3, 3)	(2, 2)
7	Concatenate_1	(73, 73, 64)	(73, 73, 160)	None	None
		(73, 73, 96)			
		Block 1 module 2			
8	Conv2d_5	(73, 73, 160)	(73, 73, 64)	(1, 1)	(1, 1)
9	Conv2d_6	(73, 73, 64)	(71, 71, 96)	(3, 3)	(1, 1)
10	Conv2d_7	(73, 73, 160)	(73, 73, 64)	(1, 1)	(1, 1)
11	Conv2d_8	(73, 73, 64)	(73, 73, 64)	(7, 1)	(1, 1)
12	Conv2d_9	(73, 73, 64)	(73, 73, 64)	(1, 7)	(1, 1)
13	Conv2d_10	(73, 73, 64)	(71, 71, 96)	(3, 3)	(1, 1)
14	Concatenate_2	(71, 71, 96)	(71, 71, 192)	None	None
		(71, 71, 96)			
		Block 1 module 2			
15	Average_pooling2d	(71, 71, 192)	(35, 35, 192)	(3, 3)	(2, 2)
16	Conv2d_11	(71, 71, 192)	(35, 35, 192)	(3, 3)	(2, 2)
17	Concatenate_3	(35, 35, 192)	(35, 35, 384)	None	None
		(35, 35, 192)			
		Block 2			
18	Conv2d_12	(35, 35, 384)	(35, 35, 32)	(1, 1)	(1, 1)
19	Conv2d_13	(35, 35, 384)	(35, 35, 32)	(1, 1)	(1, 1)
20	Conv2d_14	(35, 35, 32)	(35, 35, 32)	(3, 3)	(1, 1)
21	Conv2d_15	(35, 35, 384)	(35, 35, 32)	(1, 1)	(1, 1)
22	Conv2d_16	(35, 35, 32)	(35, 35, 48)	(3, 3)	(1, 1)
23	Conv2d_17	(35, 35, 48)	(35, 35, 64)	(3, 3)	(1, 1)
24	Concatenate_4	(35, 35, 32)	(35, 35, 128)	None	None
		(35, 35, 32)			
		(35, 35, 64)			
25	Conv2d_18	(35, 35, 128)	(35, 35, 384)	(1, 1)	(1, 1)
26	Add	(35, 35, 384)	(35, 35, 384)	None	None
		(35, 35, 384)			
		BN + ReLU			
27	Average_pooling2d	(35, 35, 384)	(4, 4, 384)	None	(1, 1)
		Dropout			
		Softmax			

### Fus2Net training and hyperparameter tuning

To speed up the training and convergence speed of Fus2Net, we perform batch normalization (BN) on the convolutional layer using the ReLU activation function [28]. After the first fully connected layer, dropout with a probability of 0.5 [29] and L2 regularization with a regularization factor of 0.05 [30] are used.

As the optimizer of the backpropagation algorithm in Fus2Net, we tried to use three optimization algorithms: Adam [31], RMSprop [32], and SGD [33]. During training, all optimizers used default parameters. The loss function used binary logistic regression with cross-entropy loss. The data were input into Fus2Net in batch mode, and the batch size was set to 16. To select the model with the best performance, we perform tenfold cross-validation on the training data. As a criterion for stopping training, 53 epochs were performed for each fold. The cross-validation method adopts the form of hierarchical grouping so that the proportion of each category in each group is as same as the proportion of each category in the overall data. Compared with dividing ten groups directly, this method overcomes the imbalance of batch data.

### Performance metric

In this study, accuracy, sensitivity, specificity, and AUC values were used as performance evaluation metrics. Accuracy is the probability of being correctly identified in all cases. The sensitivity indicates that the missed diagnosis rate is low, that is, the probability that a malignant tumor is diagnosed as malignant, and a patient whose breast tumor is malignant has not been spared. The specificity indicates that the misdiagnosis rate is low, that is, the probability that a benign tumor is diagnosed as benign, and a patient whose breast tumor is benign has not been spared. AUC, the area under the receiver operating characteristic (ROC) curve, is a common metric used to evaluate the pros and cons of a binary classification model. Generally, the higher the AUC value, the better the effect of the model:

2 $$\begin{aligned}&\text{Accuracy} = \frac{TP + TN}{TP + FP + FN + TN}, \end{aligned}$$

3 $$\begin{aligned}&\text{Sensitivity} = \frac{TP}{TP + FN}, \end{aligned}$$

4 $$\begin{aligned}&\text{Specificity} = \frac{TN}{FP + TN}, \end{aligned}$$

5 $$\begin{aligned}&\text{Precision} = \frac{TP}{TP + FP}, \end{aligned}$$

6 $$\begin{aligned}&\text{AUC} = roc\_auc\_score\left( y\_true, y\_scores\right) . \end{aligned}$$

In these equations, *TP*, *TN*, *FP*, and *FN* represent true positive, true negative, false positive, and false negative, respectively. $y\_scores$ is the probability of the predicted category, $y\_true$ is the true label of the category, and $roc\_auc\_score$ is the calculation method of AUC value.

## Data Availability

The datasets analyzed during the current study are available from the corresponding author on reasonable request.

## Abbreviations

CNN: Convolutional Neural Network;
CAD: Computer-aided diagnosis;
BUS: Breast ultrasound;
AUC: Area under curve;
ARD: Automatic relevance detection;
DL: Deep learning;
CNNs: Convolutional Neural Networks;
TL: Transfer learning;
BN: Batch normalization;
ROC: Receiver operating characteristic
